# New strategy for osseodensification during osteotomy in low-density bone: an in vitro experimental study

**DOI:** 10.1038/s41598-023-39144-z

**Published:** 2023-07-24

**Authors:** Raphael Bettach, Gilles Boukhris, Piedad N. De Aza, Eleani Maria da Costa, Antonio Scarano, Gustavo Vicentis Oliveira Fernandes, Sergio Alexandre Gehrke

**Affiliations:** 1 Associate Professor et Department of Cariology and Comprehensive Care, New York University, New York, NY 10010 USA; 2 Paris, France; 3 Instituto de Bioingenieria, Universidad Miguel Hernández de Elche, Alicante, Spain; 4 Department of Materials Engineering, Pontificial Catholic University of Rio Grande do Sul, Porto Alegre, Brazil; 5 Department of Innovative Technologies in Medicine and Dentistry, University of Chieti-Pescara, 66100 Chieti, Italy; 6 Department of Research, Bioface/PgO/UCAM, Calle Cuareim 1483, 11100 Montevideo, Uruguay; 7 Periodontics and Oral Medicine Department, University of Michigan School of Dentistry, Ann Arbor, MI 48109 USA; 8 Department of Biotechnology, Universidad Católica de Murcia (UCAM), Murcia, Spain

**Keywords:** Biotechnology, Health care, Medical research

## Abstract

The goal of this in vitro study was to evaluate and propose a new strategy for osseodensification technique using a drill counterclockwise to densification of bone of low density. Synthetic bone blocks of two different low densities (type III and IV) were used for the tests. The conventional drilling group (CD group) used Turbo-drill in a clockwise direction, and the osseodensification group (OD group) applied Turbo-drill in a counterclockwise direction. The applied tests were: (i) measurement of the temperature variation (ΔT) and (ii) measurement of the torque during the osteotomies, comparing the new strategy with the conventional drilling. Both groups were tested without (condition c1) and with (condition c2) irrigation, generating four subgroups: CDc1, CDc2, ODc1, and ODc2. Twenty osteotomies were made for each subgroup with a thermocouple positioned intra-bone (1 mm distant from the osteotomy) to measure the temperature produced. Other 20 samples/group were used to measure the torque value during each osteotomy in both synthetic bone density blocks. The mean of the ΔT during the osteotomies in type III bone was: 6.8 ± 1.26 °C for the CDc1 group, 9.5 ± 1.84 °C for the ODc1, 1.5 ± 1.35 °C for the CDc2, and 4.5 ± 1.43 °C for ODc2. Whereas, in the type IV bone, the ΔT was: 5.2 ± 1.30 °C for the CDc1 group, 7.0 ± 1.99 °C for the ODc1, 0.9 ± 1.05 °C for the CDc2, and 2.7 ± 1.30 °C for ODc2. The maximum torque during the osteotomies was: 8.8 ± 0.97 Ncm for CD samples and 11.6 ± 1.08 Ncm for OD samples in the type III bone; and 5.9 ± 0.99 Ncm for CD samples and 9.6 ± 1.29 Ncm for OD samples in the type IV bone. Statistical differences between the groups were detected in tests and conditions analyzed (*p* < 0.05). Using the drill counterclockwise for osseodensification in low-density bone generated a significantly greater torque of a drill than in CD and temperature variation during osteotomies. However, the temperature range displayed by the OD group was below critical levels that can cause damage to bone tissue.

## Introduction

Endosteal implants' use to rehabilitate missing teeth has become widely used in modern dentistry, mainly due to their predictability and long-term results^1–3^. With the advancement of biological knowledge of processes related to osseointegration of implants, new techniques have emerged to help and/or enable the use of implants in areas with some type of bone deficiency, whether in volume or density. Frequently, bone ridges healed after tooth loss are characterized by low density due to the lack of internal stimuli for a period, which may make the initial stabilization of a dental implant difficult. To better enable the initial stability of implants in those areas, some techniques have been proposed and applied, such as under-drilling^4^, the technique of manual osteotomes, manual compactors^5^, and, more recently, mechanized osseodensification^6–8^.

The osseodensification technique with rotary instruments was proposed as an alternative to other techniques, being able to compact and/or expand the bone tissue in a less traumatic way and with greater precision^7^. The osseodensification effect is due to the drill design. It presents many faces and a negative cutting angle, possibly increasing bone density while expanding the bone tissue during osteotomy^8^. Thus, these drills' design promotes compaction of the bone tissue, increasing its density laterally and, apically, improving the initial stability of the implant^4–8^. This fact can be observed in preclinical and clinical studies, which showed favorable results after applying the technique^7,9,10^.

On the other hand, the drills for osseodensification have a universal design, and their use is adapted according to the design (macrogeometry) of each implant system^11,12^. It can interfere negatively with the initial stability values. In this sense, using instruments manufactured with an adjusted design, which corresponds to the implant, may be more appropriate^12^.

However, since ossedensification drills are not sharp, irrigation during drilling must be abundant not to generate excessive heating^13^. High-temperature variation during osteotomy can cause undesirable bone tissue effects, possibly triggering peri-implantitis and implant loss (without osseointegration). Several factors are related to the increase in temperature during the osteotomy, such as drilling depth, drill design and sharpening, bone density, drilling speed, manual pressure applied, intermittent or continuous movements, and irrigation^14–17^. Gehrke et al.^17^ recently showed that bone healing is directly related to the trauma generated during the osteotomy and implant insertion torque^18,19^. The maintenance of adequate temperature levels can be achieved mainly with irrigation. This point has been approached by some companies that have developed mechanisms to improve and make the drills cooler during bone preparation, such as creating a specific device coupled to the drill, which is accelerated when the drill is activated^20^. However, adequate temperatures can be achieved via using low rotation speeds^21^. Moreover, as recently demonstrated by Achour et al.^21^, the use of drills with adequate cutting and speed can make a big difference for the collection of bone particles and subsequent use of this tissue to be used as material to fill gaps during the installation of implants.

The osseodensification technique using rotary instruments considered relatively recent, has few studies assessing the temperature variation during the procedure and no evidence of the ideal drill design^22^. Thus, the purpose of the present study was to evaluate the effects on the temperature and torque of a drill for osteotomy rotating counterclockwise to perform osseodensification. This in vitro study used blocks of synthetic low-density bone (types III and IV). Temperature variation during osteotomy (with and without irrigation) and maximum torque values during socket preparation were obtained and compared. The null hypothesis was that using the bur in a counterclockwise direction would not cause a significant increase in the local temperature variation during bed preparation for implant placement.

## Materials and methods

### Materials used

**Figure 1:**
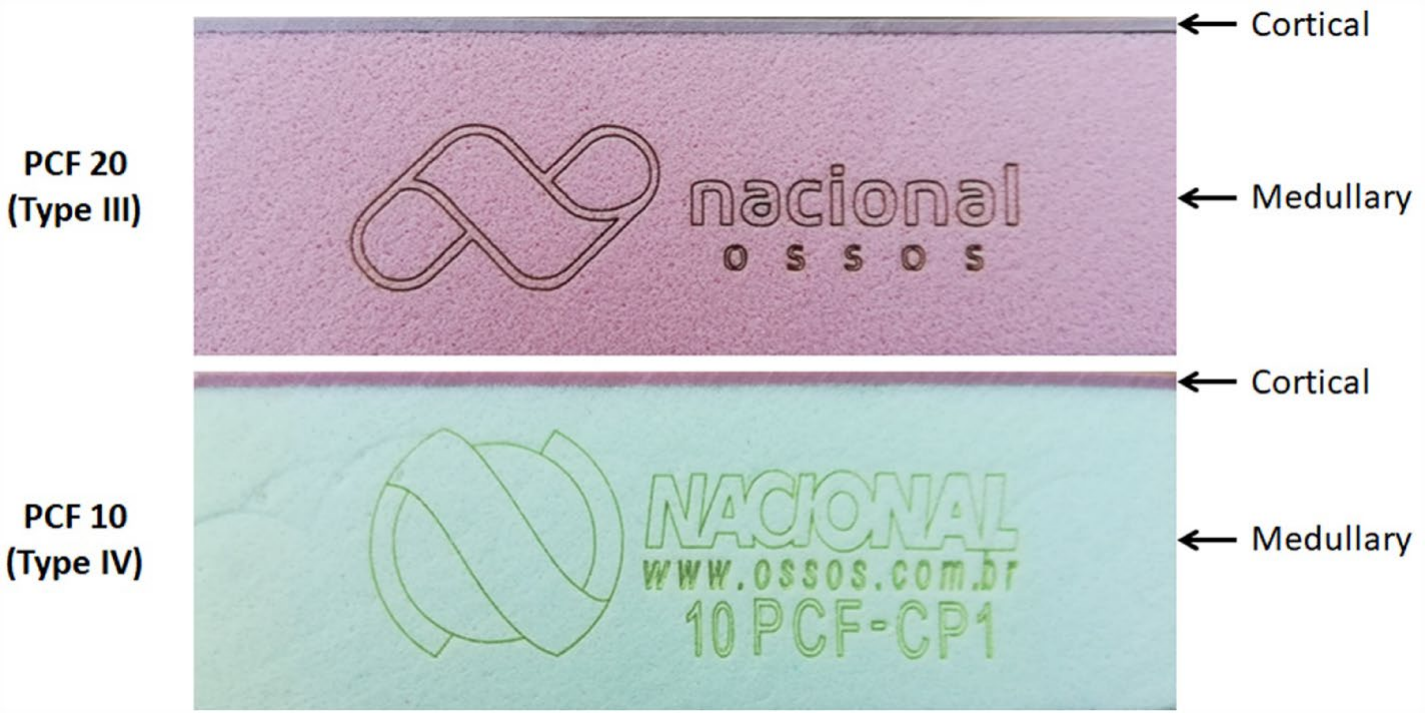
Representative image of the two synthetic bone blocks used in the present study.

Two hundred forty (n = 240) osteotomies in polyurethane foam blocks (PFB). The PFB are standardized material for testing instruments and endosseous implants by the ASTM (American Society for Testing and Materials)^23^. The PFB at 0.320 g/cm^3^ (PCF 20), simulating low bone density (bone type III), and PFB at 0.160 g/cm^3^ (PCF 10), simulating an extra-low bone density (bone type IV), were used. Both blocks with a cortical portion of 1 mm at 0.640 g/cm^3^ (PCF 40), simulating a bone density type I. The blocks' dimensions were 95 × 45 × 35 mm (Nacional Ossos, Jaú, Brazil). These block models simulating the different types of bone density were used in other recent studies published by our group^4,12^. The thermal conductivity of the standardized synthetic block is 0.3 W/mK, which is analogous to the human cortical bone (0.29 W/m/K)^24^. These thermal conductivity values assured that the measured thermal variation in this block was equivalent to those in human bone^25^. Figure 1 shows an image of both synthetic bone blocks used.

**Figure 2:**
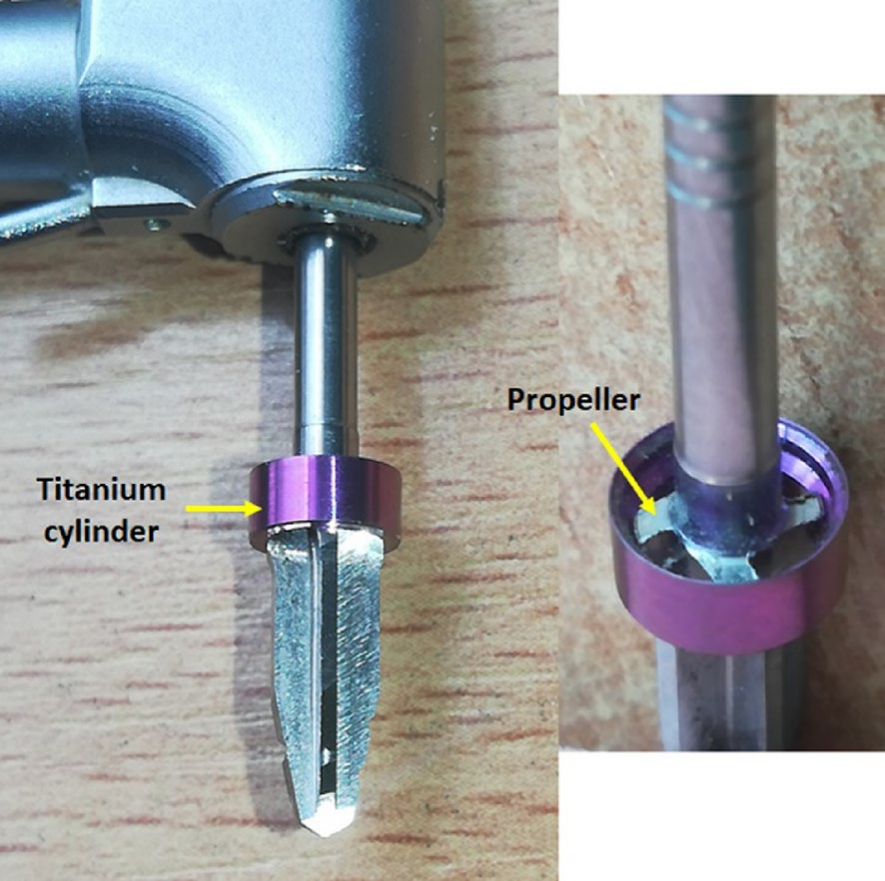
The image shows the TURBOdrill details.

All osteotomies were performed using a single drill (TURBOdrill^®^, Implant Diffusion International, Montreuil, France) for a conical implant of 4.2 × 10 mm (diameter × length) at a recommended speed of 1500 rpm. Two groups were created following the osteotomy technique performed: conventional drilling (CD group) using the TURBOdrill in clockwise rotating and osseodensification drilling (OD group) using the TURBOdrill in initial clockwise rotating until perforating the cortical bone (~ 3 mm), and counterclockwise for the rest of the osteotomy depth. Each drill was used 20 times, that is, for each situation (four subgroups, two types of synthetic bone and torque test) a new drill was used, totaling 12 drills. Figure 2 shows an image of the TURBOdrill used.

**Figure 3:**
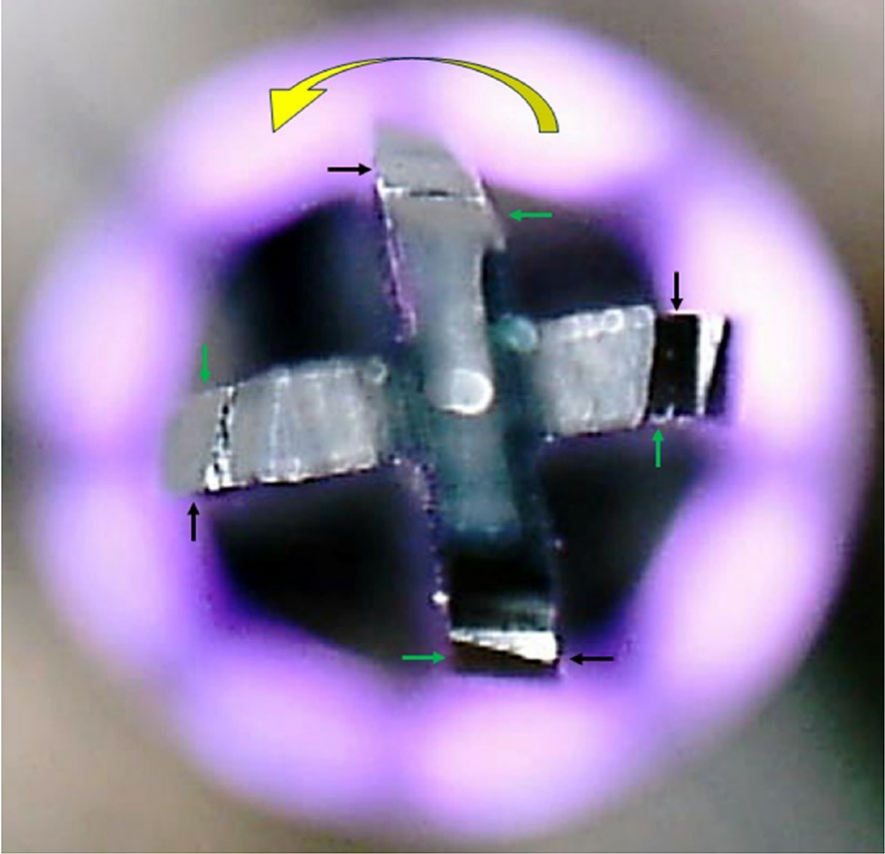
Image showing the shape of the TURBOdrill cutting blades. The black arrows show the sharp angle for clockwise cutting, and the green arrows show the more rounded, lower design of the trailing edge of the blade. Yellow arrow showing clockwise rotation direction.

Regarding the design of the cutting edges, the TURBOdrill presents an acute angle on one side of its cutting blades that rotate clockwise, a slight inclination on the edge and a slight rounding on the second edge (after the cutting edge), as shown in Fig. 3.

### Measurement of the temperature variation

Both groups were tested under two conditions. The first condition (c1) was to make the osteotomies without irrigation, and the second condition (c2) was to perform the osteotomies using an intense irrigation of 50 mL/min with distilled water at room temperature (19 ± 1 °C). Thus, four subgroups were formed for the evaluations: CDc1, CDc2, ODc1, and ODc2.

**Figure 4:**
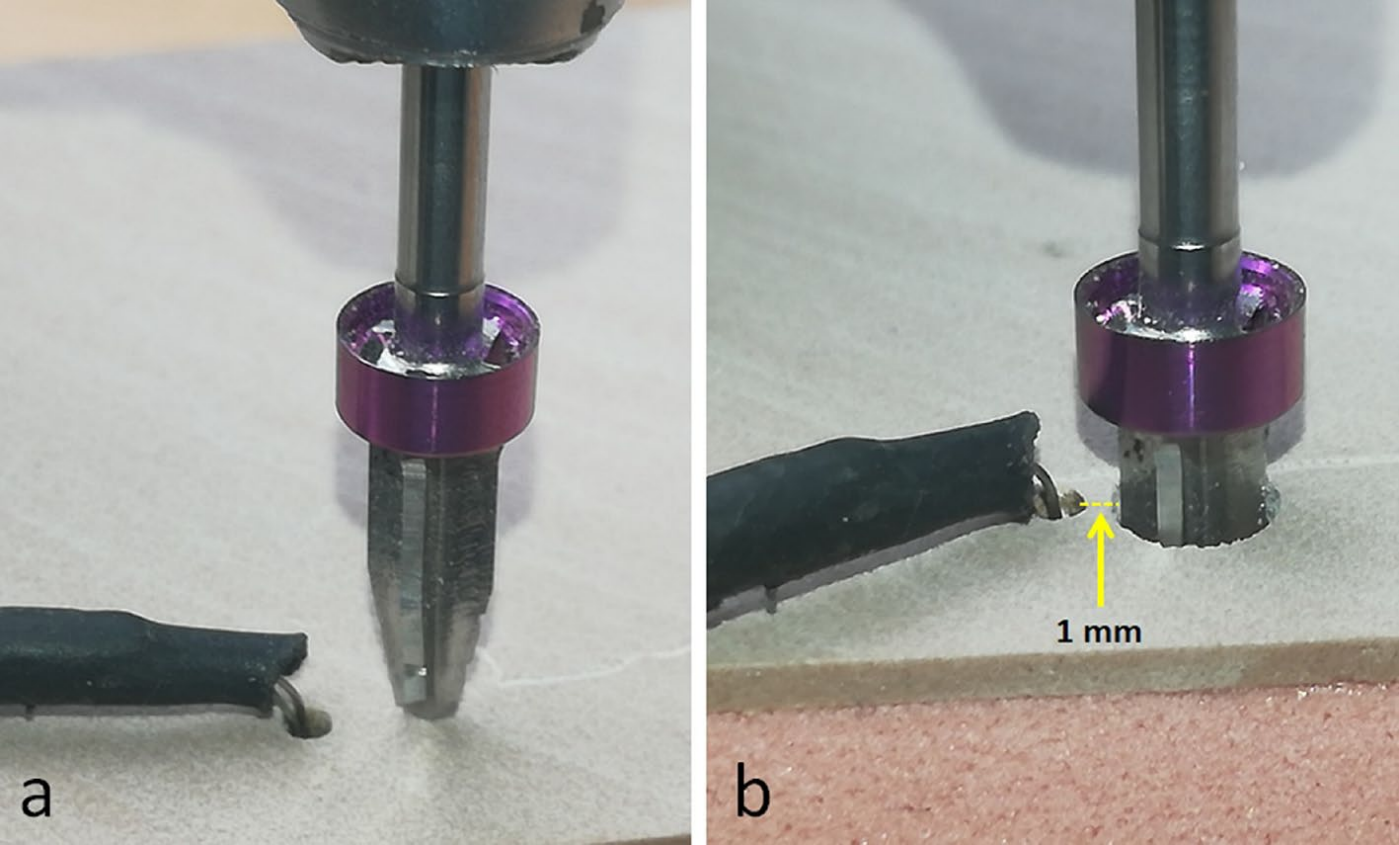
(**a**) Image of the TURBOdrill positioned to start the osteotomy and the thermocouple type k installed in the perforation. (**b**) Image after the osteotomy finished indicating the distance at 1 mm of the sensor.

For each subgroup, one synthetic bone block and one new drill were used, with a total of 20 osteotomies being performed for each subgroup. Firstly, the position of each osteotomy was marked on the model; then, we could make the lateral perforations where the 2 type K sensors (Mod. TP-01, Lutron Electronics Co., Inc., Coopersburg, PA, USA) were installed to measure the temperature during drilling. These perforations were made using a spherical carbide bur (1 mm in diameter and 2 mm in depth), calculating the distance of these perforations at 1 mm from the final diameter of the main drilling (osteotomy), as shown schematically in Fig. 4. The temperature variation depends on the density of the material, therefore the measurements were made in the cortical portion, where the density is greater and the values of temperature variation are greater, as demonstrated in a previous study^16^.

For the osteotomies, an automated system machine for drilling was used. This apparatus was used in other previous studies^14,16,20^, which permitted to control of the drilling parameters (speed, the load applied, irrigation volume, and with/without intermittent movements). The parameter values used in our study were: 2 kg of load, intermittent movements (4, 8, and 10 mm), and irrigation of 50 mL/min (in condition c2). The temperature measured before starting drilling (iT) and the maximum temperature (mT) measured during the procedure was used to calculate the temperature variation (ΔT), which was used for comparative and statistical analyses. The maximum temperature reached during the entire processor was measured (initial to the end of the drilling, plus the time that the temperature began to decrease). Also, it is important to point out that after completing each drill, the following procedure was only performed after stabilizing the room temperature value.

### Maximum torque measurement

**Figure 5:**
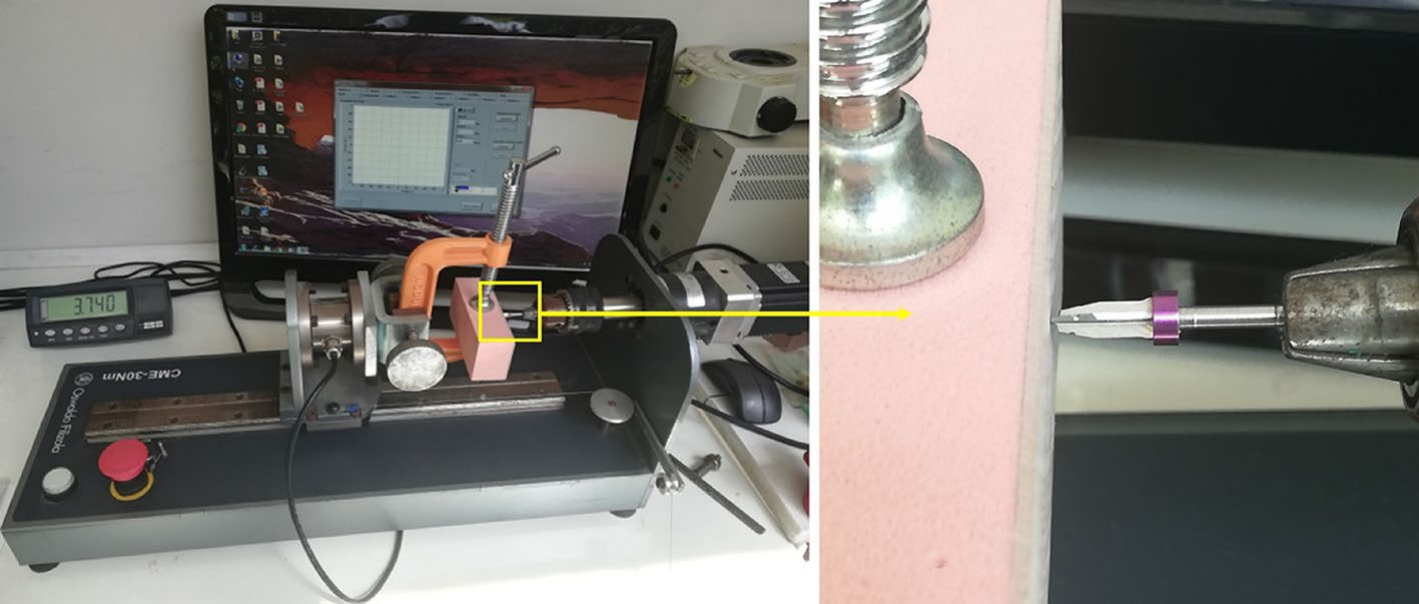
The image shows the apparatus used to measure the maximum torque during the osteotomies.

Another 80 osteotomies were performed on both density blocks (n = 20 per group) to measure the maximum torque value during drilling. For this, a computerized drilling and torque measurement apparatus CME-30 nm (Técnica Industrial Oswaldo Filizola, São Paulo, Brazil) was used, keeping the same parameters of rotation speed (1500 rpm) and load (2 kg) and intermittent movements of the test anterior, however, without irrigation. The torque test was performed only without irrigation because the test machine does not allow liquid use. Figure 5 shows an image of the osteotomies and torque measurement apparatus.

Transversal cuts were performed in 3 bone blocks of each group in both types of density, randomly selected, to verify possible differences in density after performing each osteotomy technique. Images were taken using a stereo microscope (Wild Photomacroscope M400, Wild Leitz, Heerbrugg, Switzerland).

### Statistical analysis

The data were compared statistically for both synthetic bone blocks using the ANOVA One-Way test to verify differences between the 2 groups in the 2 proposed conditions (without and with irrigation). In addition, Bonferroni's multiple comparison test was used to determine the difference between the 2 groups in the same condition and bone density in the same state but with different bone densities. GraphPad Prism version 5.01 for Windows (GraphPad Software, San Diego, CA, USA) was used to analyze the data, considering *p* < 0.05 as a statistically significant difference.

## Results

### Temperature variation

A total of 160 values of temperature variation, resulting from the difference between the maximum temperature and the initial temperature measured in each osteotomy was collected. The normality test (Kolmogorov–Smirnov test) was applied to these data, which detected a normal distribution within the groups.

**Figure 6:**
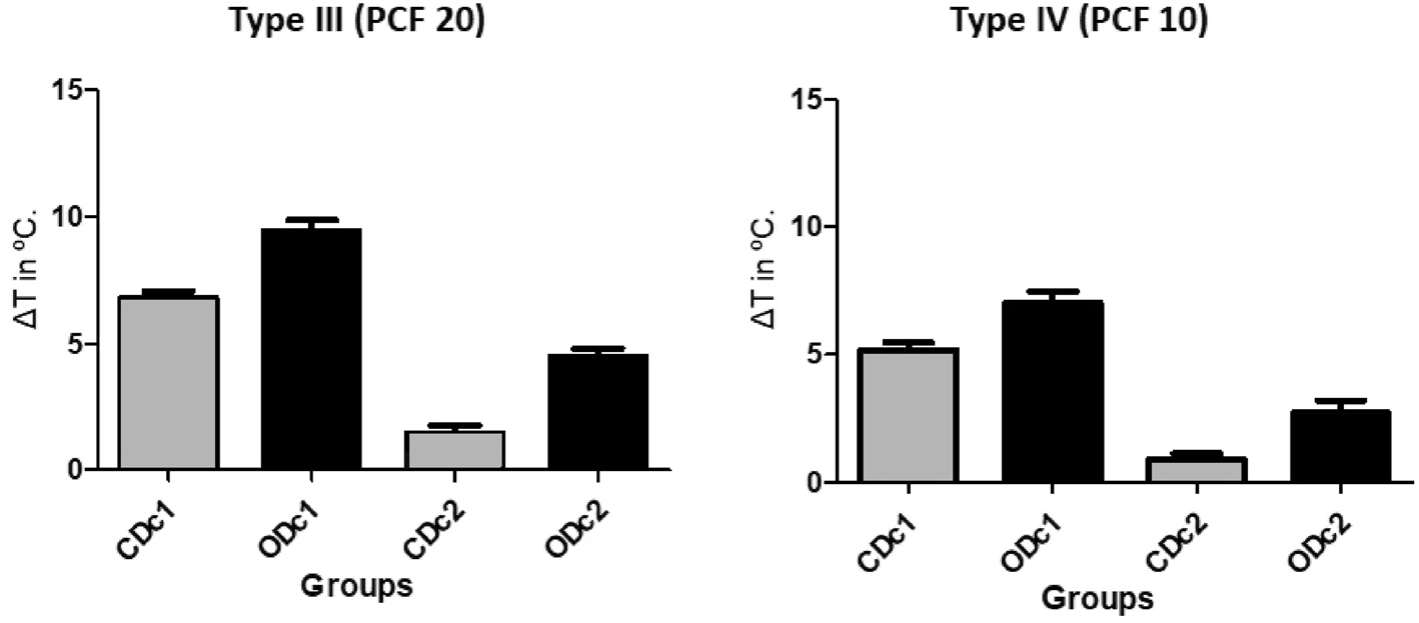
Graphic showing ΔT during the osteotomies of both groups, in the 2 conditions proposed and 2 synthetic bone blocks.

Significant differences were detected when comparing the temperature variation in osteotomies with and without irrigation within each group and in the same type of bone density (*p* < 0.0001). Whereas comparing the data obtained between the groups under the same conditions (with or without irrigation and the same type of bone density), statistically significant differences were also detected. Table 1 presents the mean values (± standard deviation), confidence interval, and intra-group and inter-group statistical comparison of temperature variation. Figure 6 shows the distribution of these data using bar graphs.

**Table 1:** Mean (± standard deviation) and confidence interval (CI) of temperature variation by groups in both bone density blocks.

Density (g/cm^3^)	CDc1 (°C)	CDc2 (°C)	ODc1 (°C)	ODc2 (°C)
Type III	6.77 ± 1.26^a^ (CI = 6.18–7.35)	1.47 ± 1.35 ^a,c^ (CI = 0.84–2.10)	9.45 ± 1.84 ^a,b^ (CI = 8.59–10.31)	4.49 ± 1.43 ^b,c^ (CI = 3.82–5.16)
Type IV	5.20 ± 1.30 ^a^ (CI = 4.59–5.80)	0.88 ± 1.05 ^a,c^ (CI = 0.39–1.37)	7.03 ± 1.99 ^a,b^ (CI = 6.10–7.96)	2.73 ± 2.05 ^b,c^ (CI = 1.77–3.69)

The same uppercase letters indicate statistically significant differences between intergroup and/or intragroup: a, b, c (*p* < 0.05).

### Maximum torque measured

**Figure 7:**
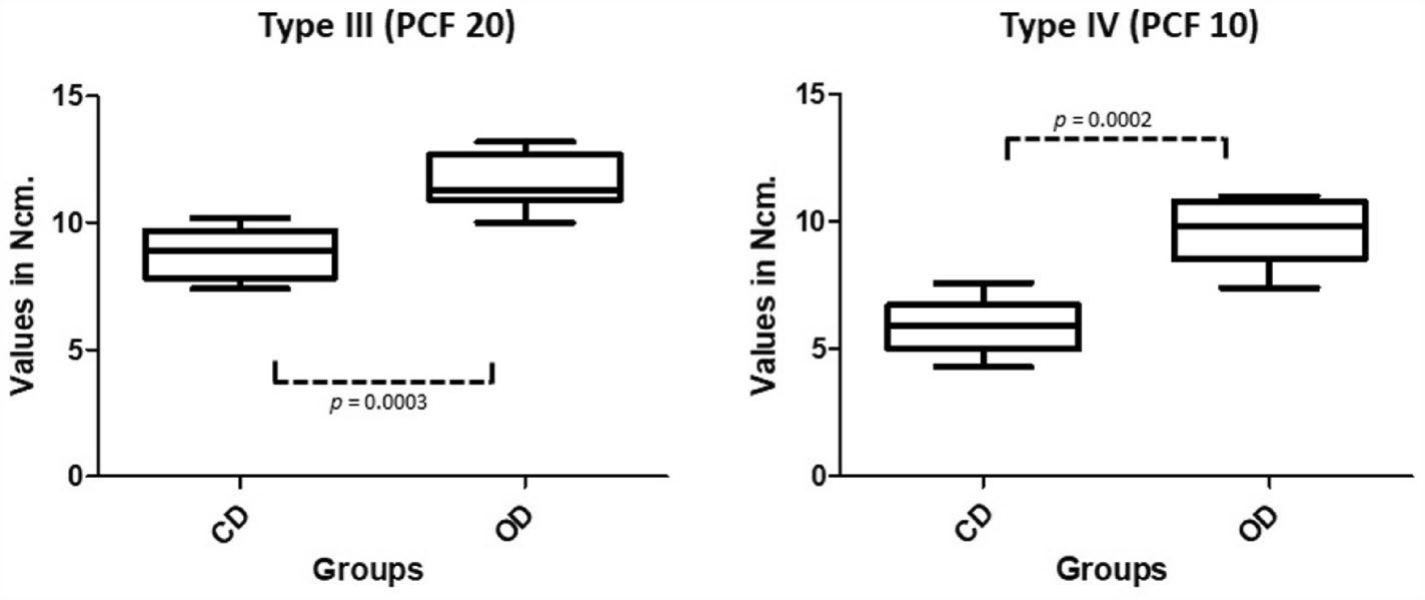
Box plots graphs showing the distribution of the maximum torque values measured during the osteotomies and the statistical comparison between both groups and the 2 synthetic bone blocks.

A total of 80 peak torque values were computed and analyzed. In both bone densities, the torque values obtained in the CD group were significantly lower than those obtained during the proposed osseodensification procedure (OD). The mean values found were: 8.8 ± 0.97 N.cm for CD samples and 11.6 ± 1.08 N.cm for OD samples in the type III density bone; and 5.9 ± 0.99 N.cm for CD samples, and 9.6 ± 1.29 N.cm for OD samples in the type IV density bone (Fig. 7). When torque values were compared between the same groups but with different bone densities (type III vs. type IV), the values also showed statistically significant differences (*p* < 0.0001).

**Figure 8:**
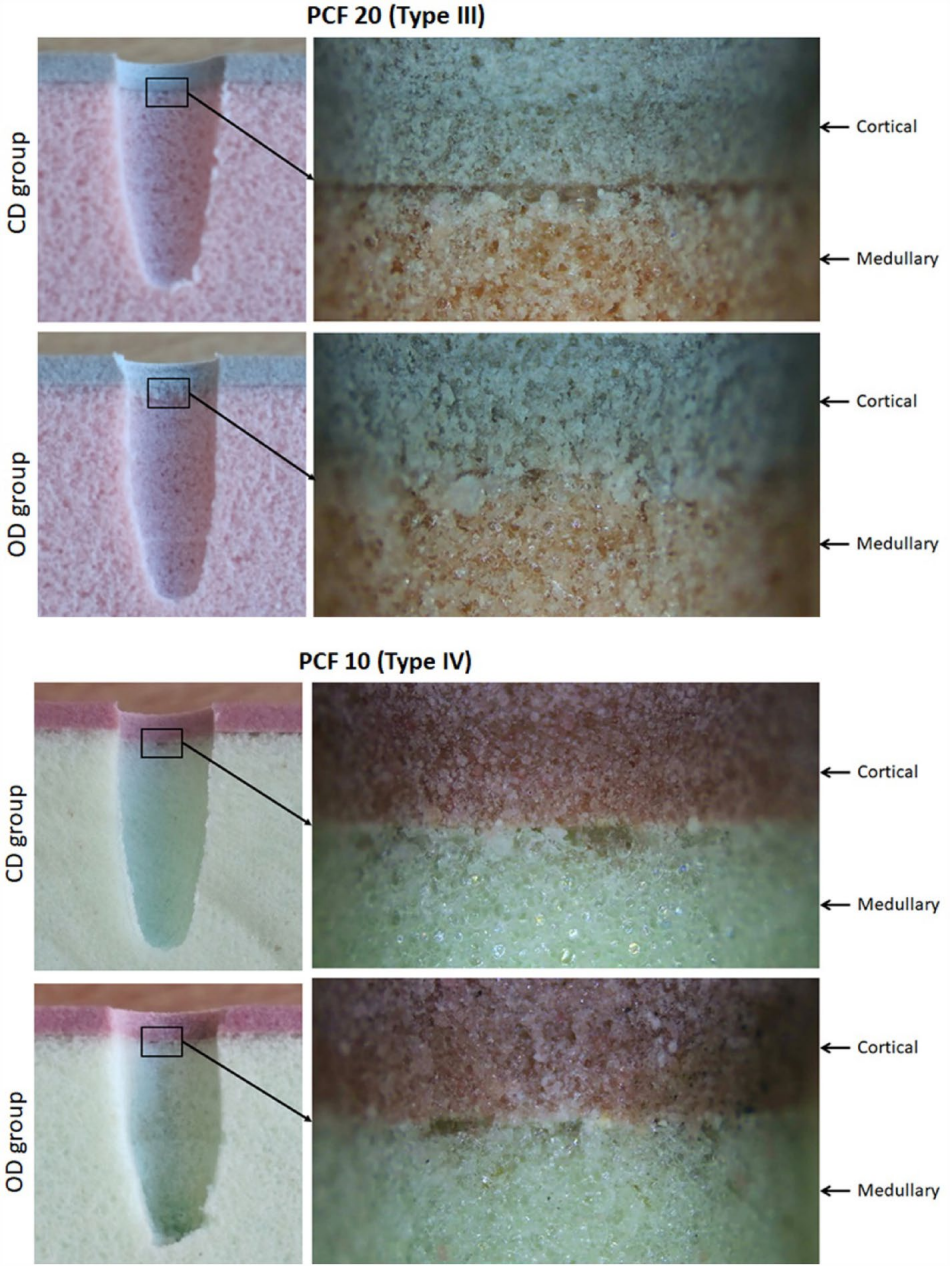
Representative images of both groups in both densities studied. In both cases was possible to observe the condensation of the medullary and cortical bone portion in the samples of the OD group compared to the CD group samples. Magnification of 10× and 100× , respectively.

In the images obtained after cross-sections the blocks at both densities, it was possible to observe the compaction of the synthetic bone in the samples from the OD group, where the bur was used in a counterclockwise direction. While in the CD group samples, where the milling cutter was used in a clockwise direction, no increase in density was observed. Figure 8 presents images of both groups at both studied densities.

## Discussion

This in vitro study aimed to assess the effects on the temperature and torque of a drill for osteotomy/densification rotating counterclockwise to promote osseodensification, with and without irrigation. Synthetic low-density bone blocks (types III and IV) were used to mimic the bones. The counterclockwise-rotation model for osteotomy preparation, known as the osseodensification technique, was designed using burs that promote lateralization of autogenous bone drilled into the surrounding cancellous structure, expanding the surrounding bone environment and increasing the local density. It can work on three different parameters: (i) it collects the bone dust and spreads them around the implant socket walls, increasing bone density and, consequently, the initial implant stability. This fact was significantly observed in our study (test groups)^26,27^; (ii) due to being a reverse drilling technique, it acts also effecting the alveolar ridge split^28^; and (iii) if used in the maxillary posterior area, the burs can push bone particles into the maxillary sinus, sinus lifting effect^29^.

The temperature variation and maximum torque were acquired using a bur rotating in a counterclockwise direction and were assessed and compared with conventional drilling in a clockwise direction. Our findings showed higher and more significant values for both parameters in the test group. Mishra & Chowdhary^30^ showed that the heat generated during the drilling process could have a multifactorial cause. Therefore, the authors suggested a drill speed of 2500 rpm with a force of 2–2.4 kg for osteotomy preparation, producing less heat. This fact was confirmed by Sharawy et al.^31^, who showed a safe drill speed at 2500 rpm with decreased risk of bony damage.

Moreover, that drill speed was evaluated for three years with reports superior of 99% for implant osseointegration, observing all bone-type densities^31^. In our study, we applied the rotation speed of 1500 rpm and a load of 2 kg in bones type III and IV, permitting a controllable temperature variance. Similar findings from Tehemar’s study^32^ stated that low hand pressure ranging around 2 kg, can be applied throughout the complete bony housing preparation with lesser heat. Moreover, the literature reported that temperatures exceeding 47 °C may result in bony necrosis due to thermal injury^27^. In concordance with that, a systematic review^33^ suggested that bone necrosis may happen in temperatures ranging between 47 and 55 °C when drilling for 1 min. Thereby, abundant irrigation is recommended, representing a simple solution for all bone drilling.

In addition, interruptions in the drilling procedure (at least every 5 s for 10 s) can dramatically decrease the chance of elevated temperatures^30^, and reusing drills more than 50 times can be another factor for bone heating and excessive damage to the tissue, impairing the osseointegration process^34^. This point was confirmed by Allsobrook et al.^35^, who suggested that drills can be used for up to 50 osteotomies without elevating temperatures and be harmful.

Regarding cell viability or better bone activity, some authors had higher and favorable results after using manual instruments or low-speed drilling (200 rpm, without irrigation) compared to the standard implant drilling process (speed > 800 rpm with copious irrigation)^36^. On the other hand, slower rotational speeds require more drilling time, which may produce more frictional heat. However, Reingewirtz et al.^37^ found a positive correlation between the temperature rise and the rotation speed. They tested a speed of 600 rpm, and it reduced the heat temperature during bone cutting and the drill speeds in dense bone.

The irrigation system, mainly using copious amounts of saline solution, has shown effective results for cooling for decades^38^. External cooling is considered the best option for cooling at superficial drill hole levels. Otherwise, in deeper holes, the internal system for cooling is a better choice. In our study, we used the traditional external system irrigation, similarly for all groups. Therefore, a combination of external and internal cooling seems beneficial, particularly in drilling compact bone sensitive to heat^14,39^.

Many methods can be used to measure the temperature generated during drilling. One of them was the real-time infrared thermography, which expresses the results by color on a monitor^40,41^; another is a shielded thermocouple, with a microprocessor thermometer recording the data obtained^31,35,37,42–44^; or using a digital thermometer for quantification of the temperature^34^, similarly used in this study. Reingewirtz et al.^37^ and Eriksson and Adell^44^ used one thermocouple with favorable results, whereas Sharawy^31^ evaluated through four thermocouples to monitor the temperature from different spots surrounding the site of drilling, which can be considered more precise.

As previously described, the conductivity values of the synthetic polyurethane blocks used in the present study present a low coefficient of thermal conductivity, which results in a heat accumulation right in front of the cutting edges^45^. Then, the sensor was installed as close as possible to the perforation site and, even at the end of each osteotomy, it was kept in position until the temperature value started to decrease.

A direct correlation was observed regarding the drilling torque tested in two different types of bone (types III and IV): the higher the bone density, the higher the drilling torque, similar to results reported in the literature^46^. Nonetheless, greater values were found for the osseodensification drilling (OD group) using the TURBOdrill counterclockwise for both bones. The torques across different bone densities indicate that the instrument responds to bone density similarly to how implant insertion torques will behave^46^. Moreover, even though the level of force can be significantly reduced with an increase in the number of revolutions of the drill due to a decrease in mean friction between the drills and the bone^47^, our study kept this variable constant.

## Conclusions

Then, it is possible to conclude that using the drill counterclockwise for osseodensification in low-density bone generated a significantly greater torque and higher temperature variation during osteotomies, rejecting the null hypothesis between conventional and osseodensification drilling. However, the temperature range displayed by the OD group was below critical levels that can cause damage to bone tissue.

## Data Availability

All data generated or analyzed during this study are included in this published article.
